# Age and Influenza-Specific Pre-Vaccination Antibodies Strongly Affect Influenza Vaccine Responses in the Icelandic Population whereas Disease and Medication Have Small Effects

**DOI:** 10.3389/fimmu.2017.01872

**Published:** 2018-01-08

**Authors:** Thorunn A. Olafsdottir, Kristjan F. Alexandersson, Gardar Sveinbjornsson, Giulia Lapini, Laura Palladino, Emanuele Montomoli, Giuseppe Del Giudice, Daniel F. Gudbjartsson, Ingileif Jonsdottir

**Affiliations:** ^1^deCODE Genetics, Amgen Inc., Reykjavik, Iceland; ^2^Faculty of Medicine, School of Health Sciences, University of Iceland, Reykjavik, Iceland; ^3^Vismederi srl, Siena, Italy; ^4^GSK Vaccines, Siena, Italy; ^5^School of Engineering and Natural Sciences, University of Iceland, Reykjavik, Iceland

**Keywords:** influenza vaccine, pre-vaccination antibody titer, age effect, underlying diseases, medication

## Abstract

Influenza vaccination remains the best strategy for the prevention of influenza virus-related disease and reduction of disease severity and mortality. However, there is large individual variation in influenza vaccine responses. In this study, we investigated the effects of gender, age, underlying diseases, and medication on vaccine responses in 1,852 Icelanders of broad age range who received trivalent inactivated influenza virus vaccination in 2012, 2013, or 2015. Hemagglutination inhibition (HAI) and microneutralization (MN) titers were measured in pre- and post-vaccination sera. Of the variables tested, the strongest association was with level of pre-vaccination titer that explained a major part of the variance observed in post-vaccination titers, ranging from 19 to 29%, and from 7 to 21% in fold change (FC), depending on the strain and serological (HAI or MN) analysis performed. Thus, increasing pre-vaccination titer associated with decreasing FC (*P* = 1.1 × 10^−99^–8.6 × 10^−30^) and increasing post-vaccination titer (*P* = 2.1 × 10^−159^–1.1 × 10^−123^). Questionnaires completed by 87% of the participants revealed that post-vaccination HAI titer showed association with repeated previous influenza vaccinations. Gender had no effect on vaccine response whereas age had a strong effect and explained 1.6–3.1% of HAI post-vaccination titer variance and 3.1% of H1N1 MN titer variance. Vaccine response, both fold increase and seroprotection rate (percentage of individuals reaching HAI ≥ 40 or MN ≥ 20), was higher in vaccinees ≤37 years of age (YoA) than all other age groups. Furthermore, a reduction was observed in the H1N1 MN titer in people ≥63 YoA, demonstrating a decreased neutralizing functionality of vaccine-induced antibodies at older age. We tested the effects of underlying autoimmune diseases, asthma and allergic diseases and did not observe significant associations with vaccine responses. Intake of immune modulating medication did not show any association. Taken together, our results show that previous encounter of influenza vaccination or infection, reflected in high HAI and MN pre-vaccination titer has the strongest negative effect on vaccine responses measured as FC and the strongest positive effect on post-vaccination titer. Increasing age had also an effect but not gender, underlying disease or medication.

## Introduction

The influenza virus causes 3–5 million cases of severe illness each year resulting in 250,000–500,000 deaths, most of which occur in elderly people [≥65 years of age (YoA)] (1). Vaccination is the best preventative measure against influenza illness and the World Health Organization (WHO) recommends annual vaccinations for high-risk groups; pregnant women, children 6 months–5 YoA, elderly individuals (≥65 YoA), individuals with chronic medical conditions and health-care workers (1). There is large individual variation in influenza vaccine responses. Factors that have been associated with impaired immune responses to influenza vaccinations include age, gender, health status of vaccine recipients, prior influenza vaccinations, and obesity (2, 3), and various immunomodulators have been reported to influence immune responses to vaccines (4–7). We therefore decided to investigate influenza vaccine responses in unselected Icelandic vaccinees of a broad age range and health conditions.

The trivalent inactivated influenza vaccine (TIV) used in this study contains hemagglutinin (HA) surface glycoprotein from two influenza A strains (H1N1 and H3N2) and one influenza B strain (either Yamagata or Victoria lineage). Vaccine-induced hemagglutin (HA) titers are widely accepted as a correlate of protection against influenza illness and are measured by the ability of HA-specific antibodies to block *N*-acetylneuraminic acid mediated viral agglutination of red blood cells using a hemagglutination inhibition (HAI) assay (8, 9). Based on this, seroprotection has been defined as HAI antibody titers ≥1:40 post-vaccination and the proportion of vaccinees achieving this titer are referred to as seroprotection rate. Following vaccination, the seroprotection rate should be >70% for adults 18–60 YoA and >60% for adults >60 YoA. In contrast to HAI titers that only measure the capacity of blocking the receptor binding of the virus to its host cell, microneutralization (MN) assays are based on the use of infectious doses of influenza virus *in vitro* thereby measuring functional antibodies that block entry of the virus into cells, fusion of the virus HA to the host cell membrane, and internalization of the virus. In addition to measuring antibodies capable of neutralizing the strain specific and immunodominant head domain, MN have been shown to detect antibodies directed to the conserved stalk of HA that could give rise to a broad protection against different strains of influenza A virus (10). Anti-stalk antibodies have been shown to be superior inducers of cytotoxicity of infected cells compared with anti-head antibodies in a mouse model and this effect was dependent on interaction with Fc receptors for IgG (FcγRs) (11). However, due to the higher cost and labor of MN compared with HAI measurements, HAI is still more widely used than MN. In this study, we measured both HAI and MN for H1N1 in all pre- and post-vaccination samples, as well as in a subset of the vaccinees (*n* = 336) for the other strains and compared the two readouts.

Due to the high-mutation rate of the influenza virus, the vaccine components need to be frequently changed to match the circulating virus strains (12). However, the potential antigenic mismatch does not account for all of the observed differences in influenza vaccine efficacy between years. In addition to reported risk factors for poor vaccine responses, variation in human leukocyte antigen (13) and other host genetic factors may play a role (14).

In addition to following WHO’s recommendations regarding the annual influenza vaccination in Iceland, many companies offer their staff annual influenza vaccinations, even if they are not in any of the risk groups. In this study, volunteers scheduled for influenza vaccination were recruited at over 70 work places and two nursing homes in the Reykjavik area when the annual influenza vaccination was offered, securing a broad age range independent of health status, and thus both risk and non-risk groups. Furthermore, we received information regarding prescribed medicines as well as diagnosis of asthma allergic and autoimmune diseases for all the study participants.

The main objective of this study was to evaluate the association of age, gender, influenza-specific pre-vaccination immune status, underlying diseases, and medication on immune responses to a seasonal TIV in unselected Icelanders of a broad age during three influenza seasons 2012–2013, 2013–2014, and 2015–2016.

We found that previous influenza virus encounter measured by high-pre-vaccination titer showed strong negative association with fold change (FC) levels whereas it showed positive association with the post-vaccination titer. Furthermore, increased age associated with vaccine responses, although to less extent than pre-vaccination titer. However, gender, underlying diseases, and immunomodulatory medication evaluated in this heterogeneous group of vaccinees did not significantly affect vaccine responses.

## Materials and Methods

### Participants and Study Design

A total of 1,852 volunteers agreed to participate in the study and were eligible during October–November in the years 2012 (*n* = 565), 2013 (*n* = 711), and 2015 (*n* = 577). Age of the vaccinees ranged from 20 to 103 YoA, 46% men and 55% women. Informed consent and pre-vaccination blood samples were obtained by staff of the Patient Recruitment Center (PRC) just before the participants received their annual influenza vaccination (administered by health-care workers) that they had signed up for either at their work places or nursing homes. Four weeks later (day 28 ± 3 days), the staff of the PRC went back to the workplaces and nursing homes to collect a post-vaccination blood sample. All participants signed informed consent. This study was carried out in accordance with the recommendations of National Bioethics Committee (NBC) of Iceland with written informed consent from all subjects. All subjects gave written informed consent in accordance with the Declaration of Helsinki. The protocol was approved by the NBC of Iceland (Approval no. VSN-12-153_VSNb2012090016-03-12). The study was reported to the Data Protection Authority of Iceland (ref. S5936/2012) that also approved access to data from the participant’s medical records (PV_2012091015T) from Landspitali, the National University Hospital of Iceland (1990–2016), and existing data at deCODE genetics. Diagnosis of autoimmune diseases we searched for included: ankylosing spondylitis, inflammatory bowel disease, multiple sclerosis, myasthenia gravis, primary biliary cirrhosis, psoriasis, psoriatic arthritis, rheumatoid arthritis, systemic lupus erythematosus, systemic sclerosis, type 1 diabetes, vitiligo, Sjögren’s syndrome, and autoimmune thyroiditis. Diagnosis of asthma and allergic diseases we searched for included: asthma, allergic rhinitis, anaphylaxis, angioedema, chronic sinusitis, nasal polyps, urticaria, and atopic dermatitis. 1,611 of the participants (87%) answered questionnaires on general health and lifestyle. Information of drug prescription for each of the participants was retrieved from the Directorate of Health Prescription Database (2003–2016) with approval of the NBC. ATC codes of drugs tested for are listed in Table S1 in Supplementary Material. The personal identities of the participants data and biological samples were encrypted using the Identity Protection System, a third-party encryption system approved, and monitored by the Icelandic Data Protection Authority.

### Vaccines

Study participants were vaccinated with Vaxigrip^®^ (Sanofi-Aventis) in the years 2012 and 2013 containing 15 μg/strain of the A/California/7/2009 (H1N1)pdm09-like virus, A(H3N2) virus antigenically like the cell-propagated prototype virus A/Victoria/361/201 (H3N2)-like virus, and the B strains: B/Wisconsin/1/2010-like virus and B/Massachusetts/2/2012-like virus strains, respectively. In 2015, the vaccine contained the same H1N1 strain together with A/Switzerland/9715293/2013 (H3N2)-like virus and B/Phuket/3073/2013-like virus.

### Clinical Samples

Serum was obtained from whole blood collected both pre-vaccination (day 0) and post-vaccination day 28 ± 3 days for serological assays. Briefly whole blood was collected in 8 ml Vacuette (Z Serum Sep Clot Activator) tubes, allowed to clot for 30 min at room temperature and kept at +4°C for up to 4 h before centrifugation at 2500 RCF við +4°C. Serum was collected, aliquoted, and kept at −80°C until analyzed by VisMederi (Siena, Italy).

### MN Assay

The MN assay was modified from a previously described procedure (15); and carried out in VisMederi laboratories. This method is based on capability of live virus to infect and replicate in cells, producing cytopathic effect (CPE) in the cell culture substrate, which is prevented by neutralizing antibodies contained in serum of vaccine subjects.

Influenza live virus A/California/07/2009 was egg propagated by VisMederi and used in the assay at the concentration of 200 TCID50/100 μl [50% tissue culture infective dose (TCID50)].

Positive and negative control sera were included in each run, as well as a back titration plate for virus titration check. In particular, antisera used were specific for each strain tested, purchased from NIBSC; depleted serum was supplied by Sigma Aldrich (Serum minus IgA/IgM/IgG, S5393).

Each heat-inactivated serum twofold diluted in microtiter plates, starting from a 1:10, was incubated with a same volume of virus solution (200TCID50/100 μl) for 1 h at 37°C and 5% CO_2_. Then 100 µl of MDCK (Madin-Darby Canine Kidney) cell suspension was added to the virus-sera mixture at the concentration of 2 × 10^5^/ml; then plates were incubated at 37°C and 5% CO_2_ for 5 days. Under optical microscope each wells was assessed for the presence of CPE (complete destruction of the cell layer in the well or the presence of holes in the cell layer, surrounded by destroyed cells), discriminating “infected” and “protected” wells. The total number of infected wells of each serum duplicate was used to calculate the MN titer of each serum sample by Spearman−Karber formula (16). Indicative seroprotection rate was defined as percentage of vaccine recipients with serum MN titer ≥20 after vaccination.

### HAI Assay

The HAI measurement was carried out in agreement with VisMederi procedures; using viral antigens, provided by NIBSC, diluted at the standard concentration of 160 hemagglutinating units (HAU)/ml and correctness of antigen dilution was checked out through a back titration in every test. Serum samples were treated with receptor destroying enzyme provided by Denka Seiken, in a ratio of 1:3, during overnight incubation at 37°C, and heat inactivated for 1 h at 56°C. Twofold serial dilutions starting from 1:10 were performed for each serum in duplicate in “V” bottomed 96-well plates. The antigen solution (4 HAU/25 μl) was added to each serum dilution and plates were incubated for 1 h at room temperature. A 0.35% solution of turkey red blood cells was added to each wells and plates were incubated for 1 h at room temperature (17). The HA protein is able to agglutinate red blood cells due to its binding affinity to surface glycoprotein of erythrocytes, and antibodies may interfere with this binding recognizing the virus antigen; this phenomenon produces an inhibition of the hemagglutination resulting in a change in the appearance of the well (18). The read out was performed by naked eye, distinguishing between the presence of hemagglutination and inhibition of it. The HAI titer was calculated as the reciprocal value of the highest serum dilution in which the hemagglutination was still inhibited. Seroprotection rate was defined as percentage of vaccine recipients with serum HAI titer ≥40 after vaccination.

### Statistical Analysis

Generalized linear regression was used to test the associations of log-transformed post-vaccination HAI or MN titers, FC, and seroprotection with various traits. The post-vaccination titer and FC were corrected for age, gender, pre-vaccination titer, year of immunization, and vaccination status (see post-vaccination titer model in Table S2 in Supplementary Material). Seroprotection was corrected for age, gender, year of immunization, and vaccination status (Table S5 in Supplementary Material). Age was split into five equal-sized groups. Vaccination status was split into three groups; one previous influenza vaccination, more than one previous influenza vaccination, and no previous influenza vaccination or information missing. To account for heteroscedasticity weighted least squares was used. Correlations between HAI and MN derived log-transformed titers were performed using linear regression. Statistical analysis was performed using the computing environment R (19).

## Results

### Overall Influenza Vaccine Responses

A total of 1,852 individuals (46% men and 54% women) at the age of 20–103 years (Table 1) that received influenza vaccination and participated in the study had both pre- and post-vaccination HAI titers for all three vaccine strains available for analysis. In addition, MN titer was measured for the whole study group for H1N1 and for a subset of 336 for H3N2 and B strains. Overall seroprotection (HAI ≥ 40) rates post-vaccination for the three study years (2012, 2013, and 2015) were 93% (H1N1), 95% (H3N2), and 65% (B strain), with the lowest seroprotection rate observed in 2015 for all three strains 90% (H1N1), 93% (H3N2), and 33% (B strain). Highest median (25th–75th quantiles) HAI post-vaccination titer across the entire study group was observed for the H3N2; 226 (113–453) followed by H1N1; 160 (80–320) and B; 40 (6–113). MN titer of 20 measured by CPE has been suggested to be predictive of protection and correspond to an HAI titer of 40 (20). Using MN ≥ 20 as definition of indicative MN seroprotection, we observed overall seroprotection rate of 63%, ranging from 60% (year 2013) up to 67 (year 2012). There was a significant difference in HAI post-vaccination titer between the three vaccination years for all three strains (H1N1 *P* = 1.4 × 10^−20^, H3N2 *P* = 0.011, B *P* = 4.7 × 10^−95^). Similarly, the H1N1 MN post-vaccination titer differed between years (*P* = 1.6 × 10^−4^, Table 1).

**Table 1:** Overview of cohort and main parameters for the influenza vaccine study in years 2012, 2013, and 2015.

Characteristics	2012	2013	2015	Whole cohort
Total subjects (%)	565 (31)	711 (38)	576 (31)	1,852
**Age**			
Mean	55	51	47	51
Median (25th–75th quantile)	55 (46–63)	50 (39–61)	47 (38–56)	51 (40–60)
Range	20–103	21–95	21–70	20–103
**Gender**			
Male (%)	188 (33)	348 (49)	307 (53)	843 (46)
Female (%)	377 (67)	363 (51)	269 (47)	1,009 (54)

**Serological vaccine response**

**H1N1 (HAI)**			
Pre-titer mean	131	115	79	109
Pre-titer median (25th–75th quantile)	57 (20–160)	67 (24–160)	40 (16–80)	57 (20–160)
Post-titer mean	319	290	180	264
Post-titer median (25th–75th quantile)	160 (80–453)	190 (113–320)	160 (80–160)	160 (80–320)
Fold increase mean	12	10	9	10
Fold increase median (25th–75th quantile)	2 (1–8)	2 (1–6)	2 (1–4)	2 (1–6)
Seroprotection rate pre-vaccination	63	70	65	67
Seroprotection rate post-vaccination	94	96	90	93
**H3N2 (HAI)**			
Pre-titer mean	135	155	54	117
Pre-titer median (25th–75th quantile)	80 (14–160)	80 (40–190)	40 (5–80)	57 (20–160)
Post-titer mean	366	436	280	366
Post-titer median (25th–75th quantile)	320 (160–640)	320 (160–640)	160 (80–320)	226 (113–453)
Fold increase mean	16	13	17	15
Fold increase median (25th–75th quantile)	3 (1–8)	2 (1–8)	4 (2–16)	3 (2–11)
Seroprotection rate pre-vaccination	62	78	52	65
Seroprotection rate post-vaccination	94	98	93	95
**B (HAI)**			
Pre-titer mean	68	24	12	34
Pre-titer median (25th–75th quantile)	40 (10–80)	5 (5–28)	5 (5–5)	5 (5–40)
Post-titer mean	225	66	29	103
Post-titer median (25th–75th quantile)	160 (80–320)	40 (13–80)	5 (5–40)	40 (6–113)
Fold increase mean	11	7	4	7
Fold increase median (25th–75th quantile)	4 (1–8)	2 (1–8)	1 (1–3)	2 (1–8)
Seroprotection rate pre-vaccination	51	24	11	28
Seroprotection rate post-vaccination	88	72	33	65
**H1N1 (MN)**			
Pre-titer mean	24	19	23	22
Pre-titer median (25th–75th quantile)	14 (7–28)	10 (7–20)	14 (7–28)	14 (7–28)
Post-titer mean	54	44	47	48
Post-titer median (25th–75th quantile)	28 (14–57)	28 (14–56)	28 (14–56)	28 (14–56)
Fold increase mean	4	3	3	3
Fold increase median (25th–75th quantile)	2 (1–4)	1 (1–4)	2 (1–2)	1 (1–4)
Seroprotection rate pre-vaccination	32	27	31	30
Seroprotection rate post-vaccination	67	60	61	63

### Previous Humoral Influenza Virus Immunity Strongly Affects Influenza Vaccine Responses

**Figure 1:**
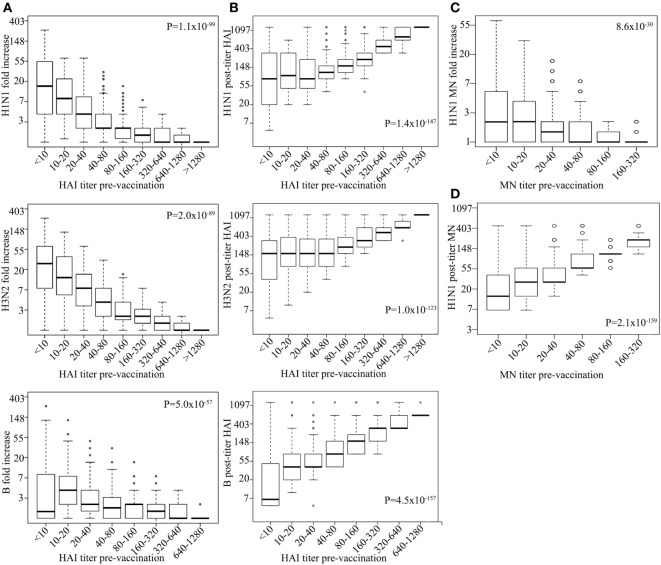
High-pre-vaccination titer strongly decreases antibody response upon influenza virus vaccination. Boxplot showing log hemagglutination inhibition (HAI) fold increase for H1N1, H3N2, and B (*y*-axes) vs. corresponding pre-vaccination titer (on *x*-axes) **(A)**. Log H1N1, H3N2, and B HAI post-vaccination titer (*y*-axes) vs. corresponding pre-vaccination titer (*x*-axes) **(B)**. Log H1N1 microneutralization (MN) fold increase (*y*-axes) vs. pre-vaccination MN titer (*x*-axes) **(C)** and log H1N1 MN post-vaccination titer vs. H1N1 pre-vaccination titer **(D)**. Line within the boxplots indicate median value and the top and the bottom correspond to the 25th (Q1) and 75th (Q3) quantiles. The whiskers of the box plots are located at Q1 − 1.5 interquartile range (IQR) and Q3 + 1.5 IQR. *P*-values for association between fold change and pre-vaccination titer **(A,C)** and post-vaccination titers and pre-vaccination titer **(B,D)** are shown. Age, measurement date, previous influenza vaccination status, and gender were included as covariates.

We tested the association of influenza virus-specific pre-vaccination immunity status with vaccine responses. Pre-vaccination HAI titers associated with FC of post-vaccination HAI titers for all strains tested, i.e., high-pre-HAI titer resulting in less fold increase in post-vaccination HAI titer (H1N1 *P* = 1.1 × 10^−99^, H3N2 *P* = 1.5 × 10^−89^, B *P* = 2.1 × 10^−57^; Figure 1A), although post-vaccination HAI titer itself was positively associated with pre-vaccination titer (H1N1 *P* = 1.4 × 10^−147^, H3N2 *P* = 1.0 × 10^−123^, B *P* = 4.5 × 10^−157^; Figure 1B; Table S2 in Supplementary Material). Pre-vaccination titer of HAI explains 27, 24, 19% of the variance in HAI post-vaccination titer and 21, 18, and 12% of the variance in FC for H1N1, H3N2, and B strains, respectively (Table S3 in Supplementary Material). Furthermore, FC of MN titers (H1N1) associated strongly with pre-vaccination titers (*P* = 8.6 × 10^−30^; Figure 1C). Similar association was observed for the pre-vaccination titer and FC of MN titer of the small subsets (*n* = 336); Table S4 in Supplementary Material measured for H3N2 (*P* = 1.4 × 10^−8^) and B strains (*P* = 2.6 × 10^−4^). Similar to the HAI titer, pre-vaccination MN titer was positively associated with post-vaccination MN titer (*P* = 2.1 × 10^−159^; Figure 1D) with the pre-vaccination titer explaining 29% of the H1N1 post-vaccination titer variance (Table S3 in Supplementary Material).

**Figure 2:**
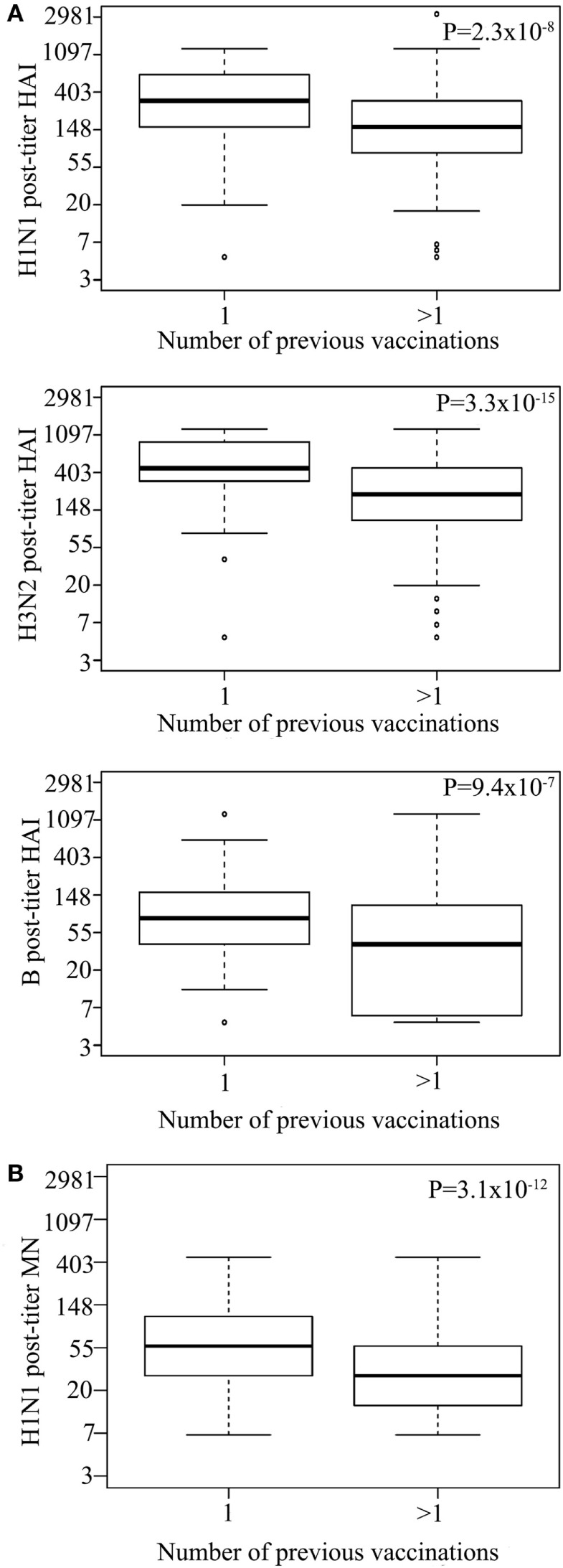
Multiple previous influenza virus vaccination associate with reduced hemagglutination inhibition (HAI) titer. Log HAI for H1N1, H3N2, and B strain **(A)** and log H1N1 microneutralization (MN) **(B)** post-vaccination titer for vaccinees that reported to have had one previous influenza vaccination or more than one previous influenza vaccination. Line within the boxplots indicate median value and the top and the bottom correspond to the 25th (Q1) and 75th (Q3) quantiles. The whiskers of the box plots are located at Q1 − 1.5 interquartile range (IQR) and Q3 + 1.5 IQR. *P*-values for association between post-vaccination titers and previous influenza vaccinations are shown. Pre-titer, age, measurement date, and gender were included as covariates.

Vaccinees reporting to have had more than one previous influenza vaccination (*n* = 1,418) had a lower post-vaccination titer for all strains than those who reported to have only had one (*n* = 106) previous influenza vaccination [HAI for H1N1 *P* = 2.32 × 10^−8^, H3N2 *P* = 3.26 × 10^−15^, B *P* = 9.39 × 10^−7^ (Figure 2A), and MN for H1N1 *P* = 3.1 × 10^−12^; Figure 2B; Table S2 in Supplementary Material]. There was no significant difference in HAI seroprotection rate between those receiving one or more than one vaccination for H1N1 (*P* = 3.6 × 10^−1^) and H3N2 (*P* = 6.1 × 10^−1^). However, those that received more than one vaccination had reduced HAI seroprotection rate for the B strain (3.9 × 10^−3^) as well as for MN seroprotection rate for the H1N1 (4.6 × 10^−6^) (Table S5 in Supplementary Material), using 1:20 MN titer as a cut-off for seroprotection (20).

Taken together, prior influenza virus-specific antibody levels, induced upon influenza virus infection and/or vaccination, strongly affect the vaccine-induced humoral response with the pre-vaccination titer explaining roughly 19–29% of the variance in post-vaccination titer.

### Age Strongly Affects Influenza Vaccine Responses, But Gender Does Not

**Figure 3:**
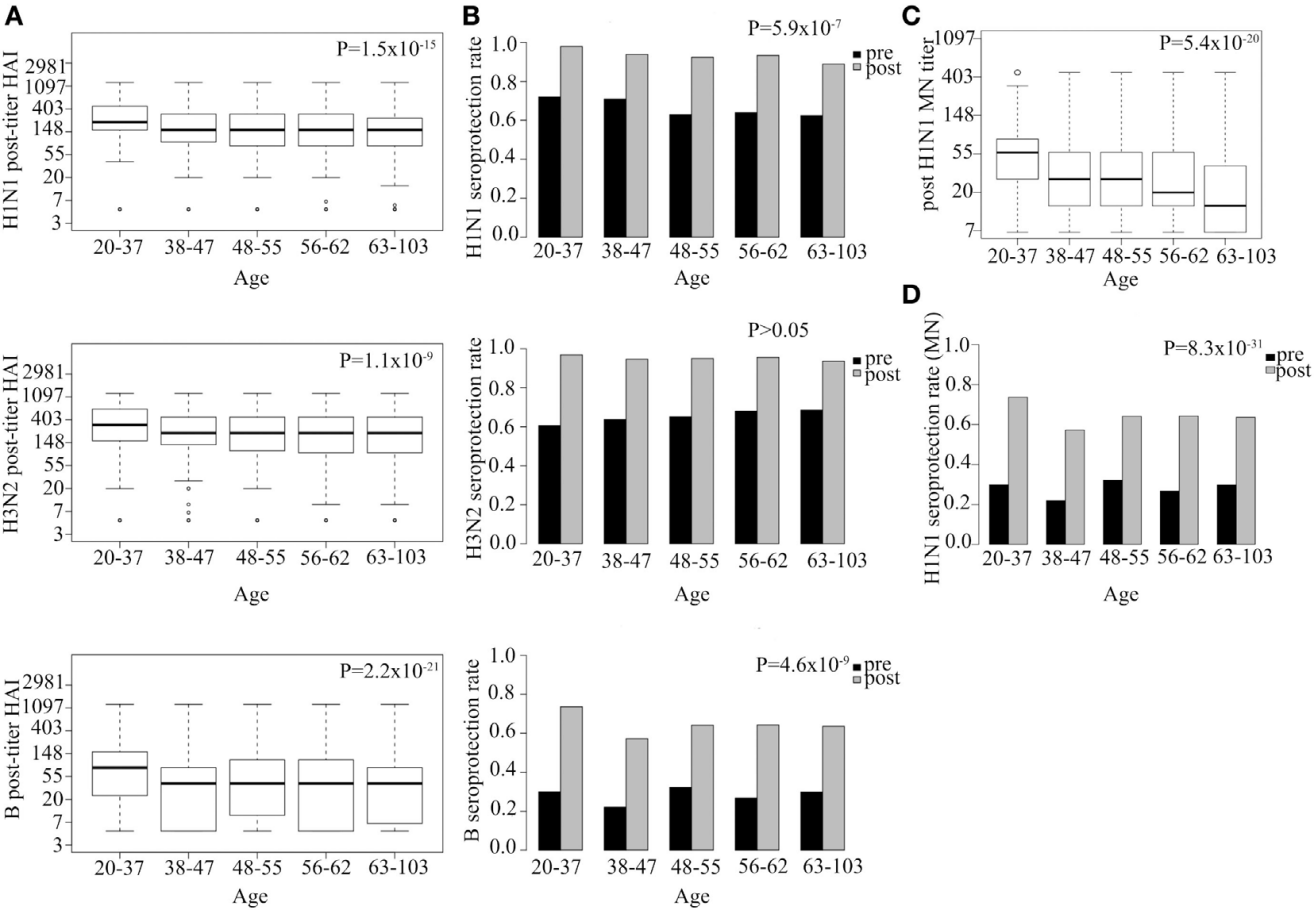
Strong association between age and both hemagglutination inhibition (HAI) and neutralization post-vaccination titer and seroprotection rate. Log HAI titer at different age gaps for all three serotypes **(A)**. HAI seroprotection rate (HAI > 40) pre- and post-vaccination at different age gaps **(B)**. Log microneutralization (MN) titer at different age gaps for H1N1 strain **(C)**. Indicative MN seroprotection rate for H1N1 (MN > 20) pre- and post-vaccination at different age gaps **(D)**. Line within the boxplots in **(A,C)** indicate median value and the top and the bottom correspond to the 25th (Q1) and 75th (Q3) quantiles. The whiskers of the box plots are located at Q1 − 1.5 interquartile range (IQR) and Q3 + 1.5 IQR. *P*-values for association between post-vaccination titer and age **(A,C)** and seroprotection rate and age **(B,D)** are shown. Pre-titer, age, measurement date, previous influenza vaccination status, and gender were included as covariates.

We tested the effect of age and gender on response to vaccination, after adjusting for pre-vaccination titers and year of immunization. Post-vaccination HAI titer went down with age for all three vaccine strains (H1N1 *P* = 1.5 × 10^−15^, H3N2 *P* = 1.1 × 10^−9^, B *P* = 2.2 × 10^−21^; Figure 3A). Age was found to explain 3, 2, and 2% of the variance in post-vaccination titers for H1N1, H3N2, B strains, respectively (Table S3 in Supplementary Material).

Hemagglutination inhibition seroprotection rate following vaccination was also reduced with increasing age for two out of three strains (H1N1 *P* = 5.9 × 10^−7^, H3N2 *P* > 0.05, B *P* = 4.6 × 10^−9^; Figure 3B). The youngest age group (20–37 YoA) had higher post-vaccination titers than any of the other age groups and the difference was most evident when compared with the oldest age group (63–103 YoA) (Table S2 in Supplementary Material).

Microneutralization titer for H1N1 also showed that increasing age associated with decrease in both post-vaccination titer (*P* = 5.4 × 10^−20^; Figure 3C) and indicative seroprotection rate (*P* = 8.3 × 10^−31^; Figure 3D), similar to what was observed for the HAI titers. Age explained 3% of the variance in H1N1 post-vaccination MN titers. Similar association was observed with age for the subset (*n* = 336; Table S4 in Supplementary Material) measured for H3N2 (*P* = 1.9 × 10^−4^) and B (*P* = 9.4 × 10^−4^) strains (data not shown). Interestingly, the effect of age on the MN titer was even more pronounced than on HAI titer indicating that the MN titer more specifically captures the age-related reduced neutralizing functionality of antibodies than the HAI titer (Figures 3A,C). No significant effects of gender on either post-vaccination HAI or MN titers or seroprotection rates were observed among all vaccinees for any of the strains tested (Figure S1 in Supplementary Material) nor among the elderly vaccinees (data not shown).

### Medication and Underlying Diseases Have Small Effects on Vaccine Responses in the Whole Group of Vaccinees

Underlying health conditions as well as medications with known immunomodulatory effects have been proposed to affect influenza vaccine responses (4–7, 21). We received data from the Directorate of Health Prescription Database on prescriptions/dispensing of various immunomodulating agents for all vaccinees from 2003 until the time of vaccination. The medication included lipid modifying agents (statins both fermented and synthetic), non-steroidal anti-inflammatory drugs, and asthma/allergy drugs (Table S1 in Supplementary Material). We used strict criteria for the prescribed medicines, dispensed 0–3 months before vaccination, to increase the likelihood of the subjects actually taking the medication at the time of vaccination. Vaccine responses did not show significant association with any of the medications studied here (Table 2). Neither was there a significant effect of any of the medications on vaccine responses among vaccinees over 65 YoA (data not shown).

**Table 2:** Effect of medication 0–3 months before vaccination on post-hemagglutination inhibition (HAI).

	H1N1 (HAI)		H3N2 (HAI)		B (HAI)		H1N1 (MN)	
Medication (*n*)	β	*P*-value	β	*P*-value	β	*P*-value	β	*P*-value
Antiparasitic products (21)	−0.026	8.9 × 10^−1^	−0.25	2.4 × 10^−1^	−0.16	4.3 × 10^−1^	0.049	7.7 × 10^−1^
Statins all (215)	−0.024	7.1 × 10^−1^	−0.075	2.9 × 10^−1^	−0.0089	9.0 × 10^−1^	0.022	7.0 × 10^−1^
Statins fermented (120)	−0.099	2.4 × 10^−1^	−0.1	2.4 × 10^−1^	0.06	5.1 × 10^−1^	−0.0082	9.1 × 10^−1^
Statins synthetic (97)	0.075	4.2 × 10^−1^	−0.018	8.5 × 10^−1^	−0.065	5.2 × 10^−1^	0.055	5.0 × 10^−1^
Biologics (32)	−0.21	1.8 × 10^−1^	−0.12	4.7 × 10^−1^	−0.26	1.2 × 10^−1^	−0.088	5.2 × 10^−1^
NSAID (144)	0.082	2.8 × 10^−1^	0.12	1.3 × 10^−1^	0.052	5.3 × 10^−1^	0.043	5.2 × 10^−1^
Asthma and Allergy (229)	−0.041	5.0 × 10^−1^	0.0076	9.1 × 10^−1^	−0.016	8.1 × 10^−1^	0.045	4.0 × 10^−1^
Inhaled corticosteroids (143)	−0.043	5.7 × 10^−1^	0.019	8.1 × 10^−1^	−0.04	6.2 × 10^−1^	0.032	6.3 × 10^−1^
Any medication (543)	−0.014	7.6 × 10^−1^	−0.0022	9.6 × 10^−1^	−0.022	6.5 × 10^−1^	0.043	2.8 × 10^−1^

We received discharge diagnosis from Landspitali, the National University Hospital of Iceland for all the vaccinees (dated from 1990), which were used in addition to the existing data at deCODE. The effect of autoimmune disease and asthma and/or allergic diseases on vaccine responses was tested. Subjects with asthma/allergic diseases had lower post-vaccination HAI titer for H3N2 strain than the rest of the vaccinees, which was nominally significant (β = −0.167, *P* = 0.024). No effects of autoimmune diseases on the post-vaccination HAI titers were observed (Table 3). Neither did autoimmune or asthma/allergic diseases affect vaccine responses among vaccinees over 65 YoA (data not shown). Self-reported general health status including information on: hospitalization due to infections, underlying diseases, and use of prescribed medicines was assessed through questionnaire data completed by 87% of vaccines. However, none of those parameters associated significantly with post-vaccination HAI titers when corrected for number of statistical tests (data not shown).

**Table 3:** Effect of underlying disease on post-hemagglutination inhibition (HAI).

	H1N1 (HAI)		H3N2 (HAI)		B (HAI)		H1N1 (MN)	
Underlying disease (*n*)	β	*P*-value	β	*P*-value	β	*P*-value	β	*P*-value
Asthma and allergy (187)	−0.033	6.2 × 10^−1^	−**0.16**	**2.0 × 10^−2^**	−0.052	4.7 × 10^−1^	−0.020	9.7 × 10^−1^
Autoimmune disease (125)	−0.056	4.9 × 10^−1^	−0.14	1.1 × 10^−1^	−0.028	7.5 × 10^−1^	−0.088	2.2 × 10^−1^

*Significant values presented in bold*.

Therefore, our data indicate that for this large heterogeneous group of vaccinees of a broad age range medications and underlying diseases had no or minimal effects on influenza vaccine responses although asthma/allergic disease warrants further investigation in a controlled clinical study.

## Discussion

Here, we report seasonal influenza vaccine-induced antibody responses of Icelanders of wide age range and health status. The strongest association with vaccine-induced HAI responses was observed for the level of pre-vaccination HAI titer, thus those with high-pre-vaccination HAI titer did not show fold increase in post-vaccination HAI titer to the same extent as those with low-pre-vaccination HAI titer. The same was observed for the more functional MN titer. Large part of the FC variance (12–21%) was explained by the pre-vaccination titer. However, post-vaccination titer itself strongly associated positively with pre-vaccination titer, meaning that even if those with high-pre-vaccination titer showed less fold increase than those with low-pre-vaccination titer, their post-vaccination HAI/MN titer was overall higher. Pre-vaccination titer explained a large part of the variance in post-vaccination titer, or 19–29% depending on the viral strain and assay used for antibody measurements.

Our results on this population-based heterogeneous group of vaccinees are in line with previous reports from smaller studies showing correlation between pre-existing antibody levels to a given strain and reduced humoral immune responses upon vaccination with the same strain (22–25). The most plausible explanation as to how pre-existing strain-specific antibodies adversely affect subsequent vaccine responses is that they bind and mask viral epitopes in a vaccine containing a homologous strain, supported by the fact that high-pre-existing antibody levels also correlate with activation of fewer strain-specific plasmablasts, and vaccine-induced memory B cells (22). However, our results demonstrate that when effect on vaccine response is evaluated the readout, fold increase vs. post-vaccination titer, must be considered. The effect of lower HAI/MN titer fold increase upon repeated vaccinations on vaccine efficacy has been debated, ranging from less efficacy (26) to reduced serious influenza disease and better efficacy (27–29) reported for individuals receiving repeated influenza vaccinations. It has been proposed that antigenic distances between annual vaccine strains on one hand and the epidemic strains on the other hand might explain those discrepancies (30). Pre-existing immunity has also been shown to affect the type of antibodies induced upon vaccination with low-pre-existing immunity to H1N1 pandemic strain inducing more broadly protecting HA stalk-specific antibodies, whereas with high-pre-vaccination immunity mainly strain-specific antibodies aimed at the HA head were induced (31). We could not distinguish between stalk- or head-specific antibodies in our MN measurements and based on previous publications claiming that antibodies toward the stalk domain appear to neutralize less potently than antibodies directed to the HA head domain (32, 33) it is likely that our MN measurements are mainly picking up head-specific antibodies. Future studies addressing the role of pre-existing immunity on antibody-dependent cell-mediated cytotoxicity (ADCC) of cells infected with homologous or heterologous influenza virus strains would be highly interesting as protective role of stalk binding antibodies has been linked with FcγR-mediated ADCC (11). Our study was not designed to evaluate vaccine efficacy. However, given that almost one-third of the post-vaccination variance (both HAI and MN) is explained by the level of pre-vaccination titers in our study and the fact that the current H1N1 strain used in seasonal influenza vaccines did not change for years following the 2009–2010 pandemic despite considerable antigenic drift in the epidemic strains of this same period (34), we believe it is high time to re-evaluate whether repeated vaccinations with the same strain are beneficial for the vaccine efficacy or not. However, it should be noted that there was no difference in HAI seroprotection rate for the H1N1 and H3N2 strains between those that had received only one compared with more than one previous vaccinations, although reduced HAI and indicative MN seroprotection levels were observed for the B and H1N1 strain, respectively, despite higher post-vaccination titer for all strains. The arbitrary cut-off we used here for indicative MN seroprotection is based on a previous publication using the same cytopathic lab test for H5N1 (20), which has not been validated for other influenza virus strains and might not reflect seroprotection levels for the H1N1 strain used in this study.

Fold change is most commonly used to evaluate vaccine responses to influenza viruses and many other pathogens. Our results clearly show that FC can be misleading for evaluation of vaccine responses in vaccine/virus experienced individuals and strongly suggests that overall post-vaccination HAI or MN titers adjusted for pre-vaccination titer are more relevant and should also be considered. This is in line with new guidelines from the European medicines agency on licensing of novel influenza vaccines in Europe that emphasize the importance of quantifying functional antibodies in addition to the HA antibody response. Furthermore, the guidelines no longer rely on the pre-defined protective threshold based on serological assays (GMT increase, seroconversion, and seroprotection based on HAI ≥ 1:40), declaring that this was not the most informative approach for different subgroups of vaccinees (35). The youngest age group (20–37 YoA) had the highest HAI post-vaccination titer (adjusted for pre-vaccination titers, gender, year of immunization, and vaccination status) and seroprotection rates for all three vaccine strains. Previously, age-associated vaccine responses have primarily been reported for young (<65 YoA) vs. elderly (≥65 YoA) subjects, whereas differences in responses within the younger adult vaccination groups has not been extensively studied. Our data are in line with a recent report of significantly different influenza vaccine-induced transcriptomic responses of people older than 35 YoA compared with younger adults (36), indicating that changes in influenza vaccine responses occur much earlier in life than frequently reported. MN that measures functional antibodies seems to better capture the effect of age on vaccine responses than HAI titer, observed in a substantial decrease in the MN titer in vaccinees at 56–62 YoA, and again in those aged 63–103 years old. Still, age explains only 2–3% of the overall variance in post-vaccination titers, corresponding to 10% of what is explained by pre-vaccination antibody levels. It was recently reported that only two previous influenza vaccinations were needed to account for the entire differences in influenza vaccine responses observed between the young and elderly groups (37). However, we observed significant effects of age on vaccine responses even when we corrected for vaccination status (if subjects had received only one or more than one previous vaccination) indicating that in our study the age effect is not entirely based on vaccine history.

The strength of this study is presented by the broad age range and the fact that vaccinees were not selected into the study, but all individuals offered vaccination at two nursing homes and over 70 workplaces, including health institutions and schools, were invited to participate. The vaccinees thus represent a snapshot of the Icelandic population that gets an annual influenza vaccination, irrespective of age and health status. This, unfortunately, also means that the study is under-powered for detecting small effects in various subgroups, such as specific disease groups, but rather gives indications for interesting future research.

Overall, our data do not point toward major effects of underlying diseases and/or medication (based on prescription and dispensing within 3 months) on influenza vaccine responses. Out of the underlying diseases tested, vaccinees with diagnosed asthma and/or allergic diseases were the only ones showing a trend toward lower post-vaccination HAI titers. The effects of influenza vaccination in asthma patients has mainly been evaluated on safety and efficacy with inconsistent results that might be due to the different cohorts, vaccines, and methodology applied (38, 39).

Chronic intake of fermentation derived and synthetic statins have previously, been linked with lower influenza vaccine responses and reduced effectiveness in an elderly cohort (21, 40). Therefore, we looked at the association of synthetically and fermentation derived statins on vaccine-induced HAI post-vaccination titer but found no significant association of vaccine responses with either class of statins or both classes combined. This could be due to the low number of elderly people among our vaccinees (*n* = 261) compared with the previous study of vaccinees ≥65 YoA (*n* = 6,961) (21). We also tested the effect of statins in our ≥65 YoA vaccinees separately where 26% of the 261 subjects were on statins but we found no significant association with vaccine responses there either (data not shown). Furthermore, our analysis is based on prescribed/dispensed statins and we did not have as detailed information on statin intake as in the previous publication (daily statin intake ≥28 days before through 22 days after vaccination).

Taken together, our results show that in the whole study group influenza virus-specific pre-vaccination immune status is the major factor affecting TIV vaccine responses, followed by age. None of the other variables tested had significant effects, considering the number of tests performed, although vaccine responses in asthma/allergic patients tended to be reduced and could be an interesting future research area.

## Ethics Statement

This study was carried out in accordance with the recommendations of National Bioethics Committee (NBC) of Iceland with written informed consent from all subjects. All subjects gave written informed consent in accordance with the Declaration of Helsinki. The protocol was approved by the National Bioethics Committee (NBC) of Iceland (Approval no. VSN-12-153_VSNb2012090016-03-12). The study was reported to the Data Protection Authority of Iceland (ref. S5936/2012) that also approved access to data from the participant’s medical records (PV_2012091015T) from Landspitali, the National University Hospital of Iceland (1990–2016), and existing data at deCODE genetics. The personal identities of the participants data and biological samples were encrypted using the Identity Protection System, a third-party encryption system approved, and monitored by the Icelandic Data Protection Authority.

## Author Contributions

IJ, TO, GS, and DG designed the study. GS, KA, TO, GG, DG, and IJ analyzed and/or interpreted the data. LP, GL, and EM performed HAI and MN measurements. TO and IJ drafted the manuscript. All authors contributed to and approved the final version of the manuscript.

## Conflict of Interest Statement

TO, GS, KA, DG, and IJ are employees of deCODE Genetics/Amgen, Inc. GG is employee of GSK vaccines (Siena, Italy). LP, GL, and EM are employees of Vismederi srl.
