# Supplementary material for: Age and Influenza-Specific Pre-Vaccination Antibodies Strongly Affect Influenza Vaccine Responses in the Icelandic Population whereas Disease and Medication Have Small Effects
Source: Front Immunol. 2018 Jan 8;8:1872. doi: 10.3389/fimmu.2017.01872 (PMC5766658; doi:10.3389/fimmu.2017.01872)
Supplement: Supplementary file 1 [file Image_1.PDF]

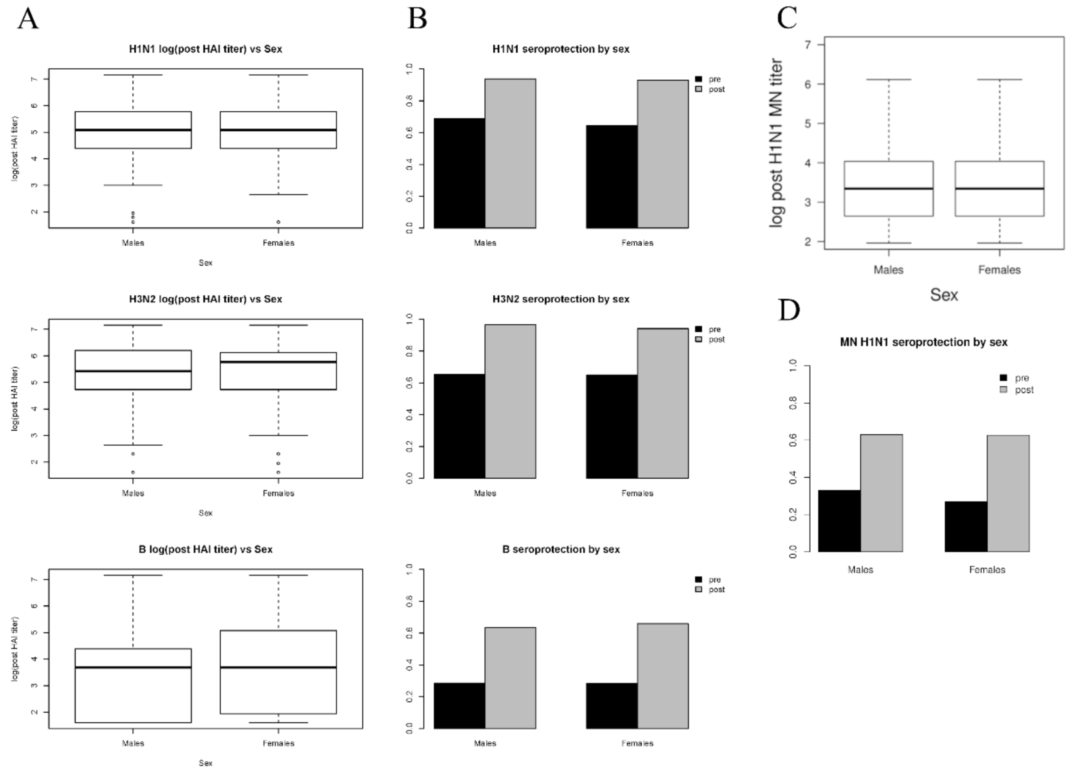

Supplementary figure 1: Gender has no effect on influenza vaccine responses. Log HAI titer vs. gender for all three serotypes for the whole study cohort (A). HAI seroprotection rate (HAI>40) pre- and post vaccination (B). Log MN titer vs. gender for H1N1 serotype for the whole study cohort (C). MN seroprotection rate (MN>20) pre- and post vaccination (D).
