# Supplementary material for: Age and Influenza-Specific Pre-Vaccination Antibodies Strongly Affect Influenza Vaccine Responses in the Icelandic Population whereas Disease and Medication Have Small Effects
Source: Front Immunol. 2018 Jan 8;8:1872. doi: 10.3389/fimmu.2017.01872 (PMC5766658; doi:10.3389/fimmu.2017.01872)
Supplement: Supplementary file 2 [file Table_1.PDF]

Supplementary Table 1: ATC codes used in the study

Medication group	ATC codes
Antiparasitic products<br>Antiparasitic products, insecticides and repellents	P01AB01, P01BA02, P01BB51, P01BC01, P01BC02, P02CA01,P02CX01
Statins all<br>Lipid modifying agents	C10AA01, C10AA02, C10AA03, C10AA05, C10AA07, C10AC01, C10AD52, C10AX09, C10BA02
Statins fermented<br>Lipid modifying agents	C10BA02, C10AA01, C10AA02, C10AA03
Statins synthetic<br>Lipid modifying agents	C10AA05, C10AA07
Biologics<br>Antineoplastic and immunomodulating agents	L01AA02, L01AB01, L01AX03, L01BA01, L01BC06, L01XX05, L04AA06, L04AA13, L04AB01, L04AB04,<br>L04AB06, L04AD01, L04AD02, L04AX01, L04AX03
Nonsteroidal antiinflammatory drugs (NSAID)<br>Antiinflammatory and antirheumatic products, non-steroids<br><br>Antiinflammatory agents, non-steroids	M01AB01, M01AB02, M01AB05 ,M01AB15, M01AB55, M01AC01, M01AC02, M01AE01, M01AE02, M01AE03, M01AE51, M01AH01, M01AH02, M01AH03, M01AH05, M01AX01, M01AX05, M01AX25, S01BC03, S01BC10
Asthma and Allergy medication<br><br>Decongestants and other nasal preparations for topical use<br>Drugs for obstructive airway diseases<br>Antihistamines for systemic use<br>Corticosteroids for systemic use, plain	R01AC01, R01AC02, R01AD01, R01AD05, R01AD08,<br>R01AD09, R01AD11, R01AD12, R03BA02, R03BA05,<br>R03BA07, R03AK06, R03AK07, R03AK10, R03AC02,<br>R03AC03, R03AC12, R03AC13, R06AA02, R06AA04, R06AA52, R06AB02, R06AD01, R06AD02, R06AD03,<br>R06AE05, R06AE07, R06AX13, R06AX22, R06AX26,<br>R06AX27, H02AA02, H02AB01, H02AB02, H02AB04, H02AB06, H02AB07, H02AB08, H02AB09
Inhaled corticosteroids (ICS)<br><br>Decongestants and other nasal preparations for topical use<br>Drugs for obstructive airway diseases	R01AD, R03BA, R03AK
