# Supplementary material for: Age and Influenza-Specific Pre-Vaccination Antibodies Strongly Affect Influenza Vaccine Responses in the Icelandic Population whereas Disease and Medication Have Small Effects
Source: Front Immunol. 2018 Jan 8;8:1872. doi: 10.3389/fimmu.2017.01872 (PMC5766658; doi:10.3389/fimmu.2017.01872)
Supplement: Supplementary file 3 [file Table_2.PDF]

Supplementary table 2. Linear regression results for association of log post-titer HAI and MN titer values for H1N1, H3N2 and B strains with relevant variables. * Compared to 2012 measurement date. ** Compared to [20,37) age group. $^{+}$ Compared to 1 previous influenza vaccination.

	H1N1 (HAI)		H3N2 (HAI)		B (HAI)		H1N1 (MN)	
Variable	β	P value	β	P value	β	P value	β	P value
Intercept	4.2	$2.1x10^{-171}$	4.8	$5.4x10^{-198}$	3.7	$4.0x10^{-118}$	2.5	$7.6x10^{-81}$
log(pre-titer)	0.43	$1.4x10^{-147}$	0.41	$1.0x10^{-123}$	0.57	$4.5x10^{-157}$	0.63	$2.1\ x10^{-159}$
Measurement date 2013*	-0.083	$9.3x10^{-2}$	0.035	$5.0x10^{-1}$	-0.79	$6.7x10^{-42}$	-0.15	$5.8x10^{-4}$
Measurement date 2015*	-0.48	$1.7x10^{-18}$	-0.13	$3.1x10^{-2}$	-1.5	$3.3x10^{-96}$	-0.18	$1.1x10^{-4}$
Age [38,48)**	-0.21	$7.5x10^{-4}$	-0.2	$3.7x10^{-3}$	-0.43	$1.3x10^{-9}$	-0.23	$2.5x10^{-5}$
Age [48,56)**	-0.3	$1.5x10^{-6}$	-0.32	$1.6x10^{-6}$	-0.41	$1.5x10^{-9}$	-0.31	$1.9x10^{-8}$
Age [56,63)**	-0.35	$9.8x10^{-8}$	-0.36	$2.8x10^{-7}$	-0.49	$1.2x10^{-11}$	-0.417.2	$1.1x10^{-12}$
Age [63,103]**	-0.55	$2.4x10^{-17}$	-0.44	$3.5x10^{-10}$	-0.71	$5.2x10^{-23}$	-0.54	$1.5x10^{-20}$
>1 previous influenza vaccinations^{+}	-0.51	$2.3x10^{-8}$	-0.79	$3.3x10^{-15}$	-0.49	$9.4x10^{-7}$	-0.54	$3.1x10^{-12}$
0 previous influenza vaccinations or NA^{+}	-0.37	$2.5x10^{-4}$	-0.62	$1.7x10^{-8}$	-0.3	$5.5x10^{-3}$	-0.42	$6.5x10^{-7}$
Sex	0.051	$2.1x10^{-1}$	0.0082	$8.5x10^{-1}$	0.011	$8.0x10^{-1}$	0.06	$9.3x10^{-2}$
