# Supplementary material for: Age and Influenza-Specific Pre-Vaccination Antibodies Strongly Affect Influenza Vaccine Responses in the Icelandic Population whereas Disease and Medication Have Small Effects
Source: Front Immunol. 2018 Jan 8;8:1872. doi: 10.3389/fimmu.2017.01872 (PMC5766658; doi:10.3389/fimmu.2017.01872)
Supplement: Supplementary file 4 [file Table_3.PDF]

**Supplementary table 3**. Percentage of variance in HAI post-vaccination titer and fold change for H1N1, H3N2 and B strains as well as MN post-vaccination titer for H1N2 explained by level of pre-vaccination titer and age.

| | H1N1 (HAI) | H3N2 (HAI) | B (HAI) | H1N1 (MN) |
|---|---|---|---|---|
| Pre-titer explaining post-titer | 27.4% | 24.2% | 19.4% | 28.6% |
| Age eplaining post-titer | 2.5% | 1.6% | 2.3% | 3.1% |
| Pre-titer explaining fold change | 21.0% | 18.4% | 11.7% | 6.5% |
| Age explaining fold change | 1.7% | 0.7% | 3.3% | 2.7% |
