# Supplementary material for: Age and Influenza-Specific Pre-Vaccination Antibodies Strongly Affect Influenza Vaccine Responses in the Icelandic Population whereas Disease and Medication Have Small Effects
Source: Front Immunol. 2018 Jan 8;8:1872. doi: 10.3389/fimmu.2017.01872 (PMC5766658; doi:10.3389/fimmu.2017.01872)
Supplement: Supplementary file 5 [file Table_4.PDF]

Supplementary table 4: Overview of the sub-cohort measured for MN titer for H3N2 and the B strain.

| Characteristics | Whole cohort |
|---|---|
| Total subjects (%) | 336 |
| Age | |
| Mean | 53,3 |
| Median (25th-75th quantile) | 54(43-62) |
| Range | 20-102 |
| Sex | |
| Male (%) | 121(36) |
| Female (%) | 215(64) |
| Serological vaccine response | |
| H1N1 | |
| pre-titer mean | 17,13 |
| pre-titer median (25th-75th quantile) | 14.14(7.07-20.00) |
| post-titer mean | 74,67 |
| post-titer median (25th-75th quantile) | 56.57(56.57-80.00) |
| Fold increase mean | 6,04 |
| Fold increase median (25th-75th quantile) | 4.00(2.00-8.00) |
| Seroprotection rate pre-vaccination | 26 |
| Seroprotection rate post-vaccination | 79 |
| B | |
| pre-titer mean | 10,51 |
| pre-titer median (25th-75th quantile) | 7.07(7.07-14.14) |
| post-titer mean | 71,99 |
| post-titer median (25th-75th quantile) | 56.57(26.21-113.14) |
| Fold increase mean | 7,55 |
| Fold increase median (25th-75th quantile) | 5.66(2.00-8.00) |
| Seroprotection rate pre-vaccination | 6 |
| Seroprotection rate post-vaccination | 75 |
