# Supplementary material for: Age and Influenza-Specific Pre-Vaccination Antibodies Strongly Affect Influenza Vaccine Responses in the Icelandic Population whereas Disease and Medication Have Small Effects
Source: Front Immunol. 2018 Jan 8;8:1872. doi: 10.3389/fimmu.2017.01872 (PMC5766658; doi:10.3389/fimmu.2017.01872)
Supplement: Supplementary file 6 [file Table_5.PDF]

Supplementary Table 5. Linear regression results for association of HAI and MN seroprotection (HAI titer values ≥ 40, MN titer values ≥ 20) for H1N1, H3N2 and B strains with relevant variables. * Compared to 2012 measurement date. ** Compared to [20,37) age group. + Compared to 1 previous influenza vaccination

	H1N1 (HAI)		H3N2 (HAI)		B (HAI)		H1N1 (MN)	
Variable	OR	P value	OR	P value	OR	P value	OR	P value
Intercept	58.39	$1.5x10^{-10}$	99.98	$4.3x10^{-9}$	51.68	$6.6x10^{-25}$	39.16	$5.8x10^{-19}$
Measurement date 2013*	1.37	$2.4x10^{-1}$	2.25	$9.5x10^{-3}$	0.28	$4.4x10^{-15}$	0.55	$2.9x10^{-6}$
Measurement date 2015*	0.45	$7.2x10^{-4}$	0.71	$1.9x10^{-1}$	0.05	$6.9x10^{-68}$	0.53	$4.3x10^{-6}$
Age [38,48)**	0.33	$8.2x10^{-3}$	0.59	$1.6x10^{-1}$	0.47	$2.5x10^{-5}$	0.51	$2.2x10^{-4}$
Age [48,56)**	0.24	$4.8x10^{-4}$	0.62	$2.1x10^{-1}$	0.47	$2.3x10^{-5}$	0.25	$2.6x10^{-15}$
Age [56,63)**	0.28	$2.6x10^{-3}$	0.75	$4.7x10^{-1}$	0.45	$2.8x10^{-5}$	0.20	$4.0x10^{-19}$
Age [63,103]**	0.13	$5.7x10^{-7}$	0.44	$3.0x10^{-2}$	0.30	$2.2x10^{-10}$	0.17	$2.6x10^{-22}$
>1 previous influenza vaccinations+	1.47	$3.6x10^{-1}$	0.73	$6.1x10^{-1}$	0.44	$3.9x10^{-3}$	0.21	$4.6x10^{-6}$
0 previous influenza vaccinations or NA+	1.52	$3.7x10^{-1}$	0.59	$4.2x10^{-1}$	0.50	$2.4x10^{-2}$	0.23	$6.2x10^{-5}$
Sex	0.84	$3.6x10^{-1}$	0.56	$1.5x10^{-2}$	0.83	$9.2x10^{-2}$	0.95	$6.0.9x10^{-1}$
